# Supplementary material for: A UMLS-based spell checker for natural language processing in vaccine safety
Source: BMC Med Inform Decis Mak. 2007 Feb 12;7:3. doi: 10.1186/1472-6947-7-3 (PMC1805499; doi:10.1186/1472-6947-7-3)
Supplement: Additional file 13 — RAP application directory. Sets up directory for RAP (RDF application for PHP) source code [file 1472-6947-7-3-S13.gz › rap/doc/phpdoc/ontModel/OntClass.html]

xml version="1.0" encoding="iso-8859-1"?


Docs For Class OntClass


## Class OntClass

Description

Description |
Methods (details)

Class that represents an ontology node characterising a class description.

History: <LI>10-05-2004 : First version of this class.</LI>

- access: public
- author: Daniel Westphal <at d-westphal dot de mail at d-westphal dot de>
- version: V0.9.1

Located in /ontModel/OntClass.php (line 24)

```
Object
   |
   --Node
      |
      --Resource
         |
         --ResResource
            |
            --OntResource
               |
               --OntClass
```

Method Summary

Description |
Methods (details)

OntClass
OntClass
([string $uri = null])

boolean
addSubClass
(object ResResource $resResource)

boolean
addSuperClass
(object ResResource $resResource)

object Individual
createInstance
([string $uri = null])

object OntClass
getSubClass
()

object OntClass
getSuperClass
()

boolean
hasSubclass
(object ResResource $resResource, [boolean $direct = true])

boolean
hasSuperClass
(object ResResource $resResource, [boolean $direct = true])

object ResIterator
listInstances
()

array
listSubClasses
([boolean $direct = true])

array
listSuperClasses
([boolean $direct = true])

boolean
removeSubClass
(object ResResource $resResource)

boolean
removeSuperClass
(object ResResource $resResource)

void
setSubClass
(object ResResource $resResource)

void
setSuperClass
(object ResResource $resResource)

Methods

Description |
Methods (details)

Constructor OntClass (line 33)

Constructor You can supply a uri

- access: public

OntClass

OntClass
([string $uri = null])

- string
  $uri

addSubClass (line 45)

Add a sub-class of this class.

- access: public

boolean

addSubClass
(object ResResource $resResource)

- object ResResource
  $resResource

addSuperClass (line 57)

Add a super-class of this class.

- access: public

boolean

addSuperClass
(object ResResource $resResource)

- object ResResource
  $resResource

createInstance (line 284)

Answer a resource that represents an instance of this OntClass and Individual node in this model.

If a resource with the given uri exists in the model, it will be re-used. If not, a new one is created in the updateable sub-model of the ontology model.

- access: public

object Individual

createInstance
([string $uri = null])

- string
  $uri

getSubClass (line 69)

Answer a class that is the sub-class of this class.

If there is more than one such class, an arbitrary selection is made.

- return: or NULL
- access: public

object OntClass

getSubClass
()

getSuperClass (line 85)

Answer a class that is the super-class of this class.

If there is more than one such class, an arbitrary selection is made.

- return: or NULL
- access: public

object OntClass

getSuperClass
()

hasSubclass (line 100)

Answer true if the given class is a sub-class of this class.

$direct - If true, only search the classes that are directly adjacent to this class in the class hierarchy.

- access: public

boolean

hasSubclass
(object ResResource $resResource, [boolean $direct = true])

- object ResResource
  $resResource
- boolean
  $direct

hasSuperClass (line 119)

Answer true if the given class is a super-class of this class.

$direct - If true, only search the classes that are directly adjacent to this class in the class hierarchy.

- access: public

boolean

hasSuperClass
(object ResResource $resResource, [boolean $direct = true])

- object ResResource
  $resResource
- boolean
  $direct

listInstances (line 135)

Answer an ResIterator over the individuals in the model that have this class among their types.

- access: public

object ResIterator

listInstances
()

listSubClasses (line 166)

Answer an array over the classes that are declared to be sub-classes of this class.

Each element of the array will be an OntClass. $direct - If true, only search the classes that are directly adjacent to this class in the class hierarchy.

- access: public

array

listSubClasses
([boolean $direct = true])

- boolean
  $direct

listSuperClasses (line 201)

Answer an array over the classes that are declared to be super-classes of this class.

Each element of the array will be an OntClass. $direct - If true, only search the classes that are directly adjacent to this class in the class hierarchy.

- access: public

array

listSuperClasses
([boolean $direct = true])

- boolean
  $direct

removeSubClass (line 229)

Remove the given class from the sub-classes of this class.

- access: public

boolean

removeSubClass
(object ResResource $resResource)

- object ResResource
  $resResource

removeSuperClass (line 241)

Remove the given class from the super-classes of this class.

- access: public

boolean

removeSuperClass
(object ResResource $resResource)

- object ResResource
  $resResource

setSubClass (line 253)

Assert that this class is super-class of the given class.

Any existing statements for subClassOf on prop will be removed.

- access: public

void

setSubClass
(object ResResource $resResource)

- object ResResource
  $resResource

setSuperClass (line 269)

Assert that this class is sub-class of the given class.

Any existing statements for subClassOf on prop will be removed.

- access: public

void

setSuperClass
(object ResResource $resResource)

- object ResResource
  $resResource

#### Inherited Methods


Inherited From OntResource

> OntResource::OntResource()  
> OntResource::addComment()  
> OntResource::addIsDefinedBy()  
> OntResource::addLabelProperty()  
> OntResource::addProperty()  
> OntResource::addRDFType()  
> OntResource::addSeeAlso()  
> OntResource::asClass()  
> OntResource::asIndividual()  
> OntResource::asOntProperty()  
> OntResource::getComment()  
> OntResource::getInstanceRdfType()  
> OntResource::getIsDefinedBy()  
> OntResource::getLabelProperty()  
> OntResource::getPropertyValue()  
> OntResource::getRDFType()  
> OntResource::getSeeAlso()  
> OntResource::getVocabulary()  
> OntResource::hasComment()  
> OntResource::hasLabelProperty()  
> OntResource::hasRDFType()  
> OntResource::hasSeeAlso()  
> OntResource::isDefinedBy()  
> OntResource::listComments()  
> OntResource::listIsDefinedBy()  
> OntResource::listLabelProperties()  
> OntResource::listProperty()  
> OntResource::listRDFTypes()  
> OntResource::listSeeAlso()  
> OntResource::removeComment()  
> OntResource::removeDefinedBy()  
> OntResource::removeLabelProperty()  
> OntResource::removeProperty()  
> OntResource::removeRDFType()  
> OntResource::removeSeeAlso()  
> OntResource::setComment()  
> OntResource::setInstanceRdfType()  
> OntResource::setIsDefinedBy()  
> OntResource::setLabelProperty()  
> OntResource::setPropertyValue()  
> OntResource::setRDFType()  
> OntResource::setSeeAlso()  
> OntResource::setVocabulary()

Inherited From ResResource

> ResResource::ResResource()  
> ResResource::addProperty()  
> ResResource::equals()  
> ResResource::getAssociatedModel()  
> ResResource::getIsAnon()  
> ResResource::getProperty()  
> ResResource::hasProperty()  
> ResResource::listProperties()  
> ResResource::removeAll()  
> ResResource::removeProperties()  
> ResResource::setAssociatedModel()  
> ResResource::setIsAnon()  
> ResResource::setURI()

Inherited From Resource

> Resource::Resource()  
> Resource::equals()  
> Resource::getLabel()  
> Resource::getLocalName()  
> Resource::getNamespace()  
> Resource::getURI()  
> Resource::toString()

Inherited From Object

> Object::toString()

Documentation generated on Thu, 7 Jul 2005 13:42:20 +0200 by phpDocumentor 1.3.0RC3
